# Complex Species Status for Extinct Moa (Aves: Dinornithiformes) from the Genus *Euryapteryx*

**DOI:** 10.1371/journal.pone.0090212

**Published:** 2014-03-03

**Authors:** Leon Huynen, David M. Lambert

**Affiliations:** Griffith School of Environment and the School of Biomolecular and Physical Sciences, Griffith University, Nathan, Queensland, Australia; University of Kent, United Kingdom

## Abstract

The exact species status of New Zealand's extinct moa remains unknown. In particular, moa belonging to the genus *Euryapteryx* have been difficult to classify. We use the DNA barcoding sequence on a range of *Euryapteryx* samples in an attempt to resolve the species status for this genus. We obtained mitochondrial control region and the barcoding region from *Cytochrome Oxidase Subunit I* (*COI*) from a number of new moa samples and use available sequences from previous moa phylogenies and eggshell data to try and clarify the species status of *Euryapteryx*. Using the *COI* barcoding region we show that species status in *Euryapteryx* is complex with no clear separation between various individuals. Eggshell, soil, and bone data suggests that a *Euryapteryx* subspecies likely exists on New Zealand's North Island and can be characterized by a single mitochondrial control region SNP. *COI* divergences between *Euryapteryx* individuals from the south of New Zealand's South Island and those from the Far North of the North Island exceed 1.6% and are likely to represent separate species. Individuals from other areas of New Zealand were unable to be clearly separated based on *COI* differences possibly as a result of repeated hybridisation events. Despite the accuracy of the *COI* barcoding region to determine species status in birds, including that for the other moa genera, for moa from the genus *Euryapteryx*, *COI* barcoding fails to provide a clear result, possibly as a consequence of repeated hybridisation events between these moa. A single control region SNP was identified however that segregates with the two general morphological variants determined for *Euryapteryx*; a smaller subspecies restricted to the North Island of New Zealand, and a larger subspecies, found on both New Zealand's North and South Island.

## Introduction

The extinct moa (Aves: Dinornithiformes) of New Zealand represented one of the fastest radiations known for birds [1]. As a result moa were comprised of a relatively large number of species that can be grouped into six genera. One of these genera, *Euryapteryx*, has been difficult to characterize into its constituent species [1]. Morphologically, *Euryapteryx* are distinguished from the other moa by a number of cranial, sternum, and leg bone features, with the current status suggesting a single species that clinally increased in size with increasing latitude and also with glacial period populations being substantially larger than their Holocene counterparts [1], [2]. At the molecular level, *Euryapteryx* are difficult to separate into distinct clades with two large scale analyses based on mitochondrial sequences suggesting two possible clades; one in the Far North of New Zealand, and one in the far south [3], [4]. Interestingly, mitochondrial control region sequences from *Euryapteryx* samples outside the far north and far south regions fail to form geographically distinct clades with samples from each island grouping together, a feature not shown by any other moa species [3], [4]. To try and resolve the species status of *Euryapteryx*, a number of individuals were tested by DNA barcoding analysis using approximately 600 bp of sequence from the 5′ terminus of the mitochondrial barcoding gene *Cytochrome Oxidase subunit I* (*COI*; [5]). *COI* barcoding of birds has been particularly successful with a concordance rate of over 96% for over 1000 species [6], [7]. Although *COI* barcode sequences were able to successfully group all recognized moa species [3], [4], [5], [8], [9] previous barcode analysis failed to clarify the species status in *Euryapteyrx* [5]. Recent work with moa eggs showed that eggshells belonging to *Euryapteryx* could be divided into two classes; a thick class (class I), and a thin class (class II), each class being associated with a specific control region sequence [10], providing evidence that separate populations at least exist in this genus. By combining the available morphological and molecular data, Worthy and Scofield (2012) [2] suggest that *Euryapteryx* consisted of two subspecies; *Euryapteryx curtus curtus*, restricted to the North Island of New Zealand, and *Euryapteryx curtus gravis*, restricted to New Zealand's South Island. To try and verify the species status of *Euryapteryx* we reanalyse the available data. In addition we determine the COI sequences for two rare, geographically important *Euryapteryx* samples as well as *Euryapteryx* samples from the Far North of New Zealand's North Island. The results obtained from these sequences provide a clearer picture of the species status of Euryapteryx and suggest that two species of *Euryapteryx* may have existed during the Holocene as well as a subspecies (possibly attributable to *E. curtus curtus*) that is found solely on New Zealand's North Island.

## Results

**Figure 1:**
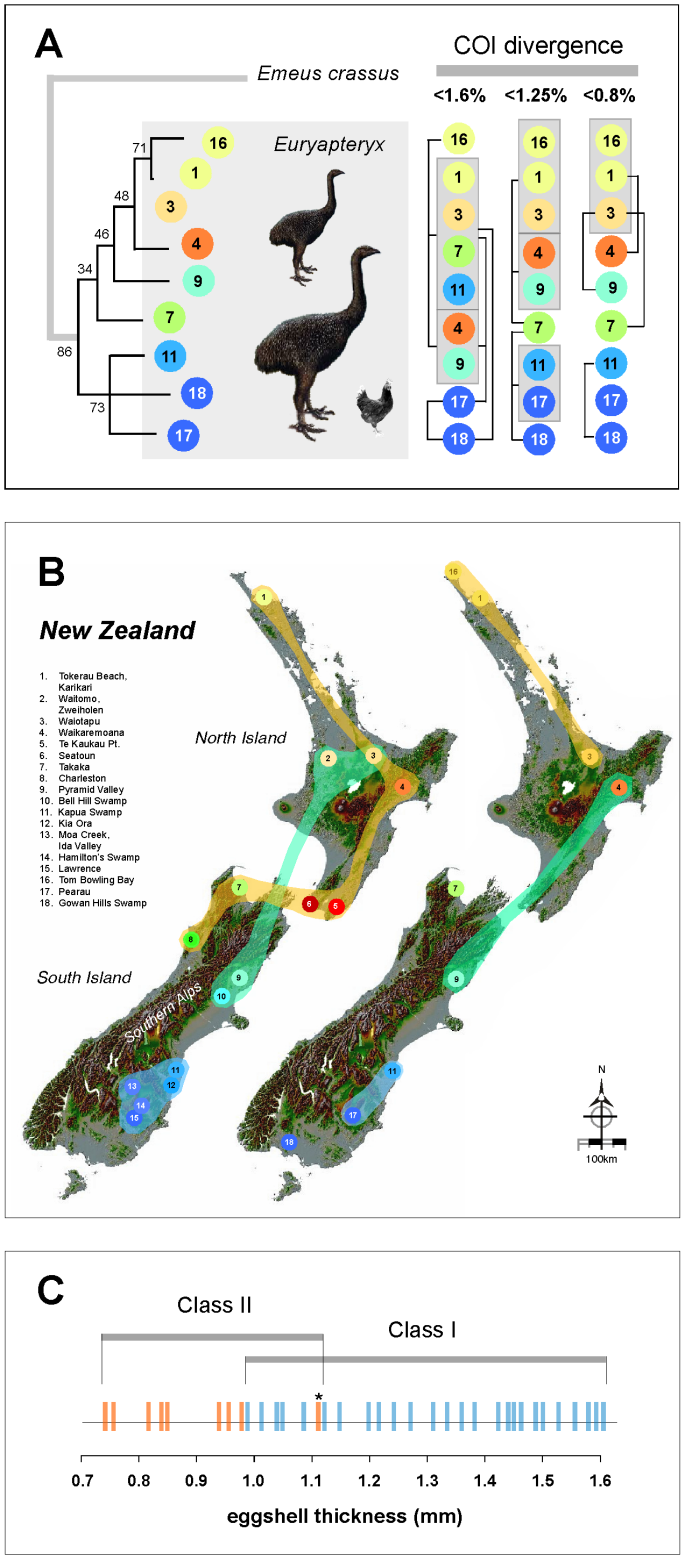
COI sequence differences, biogeography, and eggshell thicknesses of *Euryapteryx*. **A**. Phylogenetic analysis and grouping of *Euryapteryx* samples at various levels of COI sequence divergence. A phylogenetic tree was constructed in MEGA 5.05 [18] using Maximum Likelihood and Tamura-Nei parameters (log likelihood −1581.8; [20]). Bootstrap values were calculated from 500 replications. Sequence differences were calculated using K2 parameters. Individual *Euryapteryx* samples are numbered (for museum voucher numbers see supplementary information) and coloured according to location (see B). Samples are grouped according to percent COI divergence (<0.8%, <1.25%, and <1.6%). See supplementary information for divergence tables. Approximate sizes for two genetic variants (557C/T) of *Euryapteryx* (see text) are shown against that of an adult chicken. **B**. Biogeography of *Euryapteryx* populations according to (left) mitochondrial control region sequences from [4] or (right) COI sequences. Samples that form clades are joined by colour. The main COI groups were determined using a <1.25% divergence limit. This limit most closely approximated the clades formed using control region sequences. The complex interactions between individual members of each COI clade (see A) are not shown. Figure numbers refer to moa samples; 1 - AIM B6595ii [3], 2 - WO 527 [4], 3 - AIM B6580 [3], 4 - AIM B6228 [3], 5 - MNZ S40891 [4], 6 - MNZ S465 [4], 7 - CM Av21330 [3], 8 - CM Av29440a [4], 9 - CM Av8378 [3], 10 - MNZ S39965 [4], 11 - CM Av9188 [3], 12 - AM 6237 [4], 13 - OU Anthro FB271 [4], 14 - OM Av4735 [4], 15 - OM Av5191 [4], 16 - AIM B9243, 17 - OM Av9821 [3], 18 - CM Av38561 [3]. **C**. Eggshell thicknesses of *Euryapteryx*. Eggshell thicknesses from [10] (mm) are grouped according to association with class I (blue) or class II (orange) control region sequences [10]. These sequences cover a highly variable ∼30 bp fragment that is capable of distinguishing ‘thin’ *Euryapteryx* eggshells from ‘thick’. *The association of a class II sequence with this 1.11 mm eggshell may be in doubt as the sequence was obtained from the outer layer of the eggshell [10].

Mitochondrial DNA control region sequences of 389 bp and 677 bp [3], [4] suggest two defined clades for *Euryapteryx,* one each at the far northern and southern regions of New Zealand, with *Euryapteryx* samples from other locations failing to form well-supported clades [3], [4]. To try and determine the species status of *Euryapteryx* we sequenced the *COI* barcoding region for two new *Euryapteryx* samples (Table 1) and combined these with available *Euryapteryx COI* sequences such that each of the clades was represented by at least one sample (Table S1). All samples were from Holocene material. K2P distances were calculated for a number of moa including all *Euryapteryx COI* sequences (Table S2) and species cut-off limits were applied to try and delineate species (Table S3). We chose three *COI* divergence limits to try and group species; <0.8%, <1.25% (shown by [6] to be successful at determining species status in 260 North American bird taxa), and <1.6% (shown by [7] to provide the best ratio of least false negatives and least false positives for nearly 400 Palearctic bird species, and also shown to be effective at separating the five species of kiwi (*Apteryx* spp;[11], [12]) a close relative of the moa (Table S4). *COI* divergence groupings were calculated in MEGA 5.05 using K2P distance and a moa *COI* dataset of 37 sequences that included all known species ([3], [5]; Table S2). At <0.8% *COI* divergence *Euryapteryx* from the south of New Zealand's South Island and those from the far north of the North Island form two loose groups, with individuals from the latter also grouping with those from mid North Island and mid South Island (Figure 1A). At this level of divergence all moa species group as shown previously [5], [9] except for those from the genus *Anomalopteryx* where individuals from the North Island and South Island are separated by over 1% *COI* divergence (Table S2). A *COI* divergence limit of <1.25% most accurately retains the currently accepted species status for moa [4], [5] and is most similar to the geographic groupings obtained by [4] using 389 bp of control region sequence, but is also unable to fully resolve *Euryapteryx* (Figure 1B). Similar to the results obtained using the <0.8% *COI* divergence cut-off, loose groupings are found for *Euryapteryx* samples from the far north of the North Island and the south of the South Island with multiple interactions of indivuals from these groups with those from other locations (Figure 1A). At <1.6% *COI* divergence all moa species group as outlined in [5] except North Island and South Island *Dinornis* group as one species and *Pachyornis elephantopus* groups with *Pachyornis geranoides*.

**Table 1:** Moa bone samples extracted for DNA for this work.

Museum ID	bone	Location	Reference/Notes
AIM B9243	fr	Tom Bowling Bay	Collected by B. Gill, 1999.
AIM B6595ii	fr	Tokerau Beach	[3] ID - T. H. Worthy
AIM B6580	fr	Waiotapu	[3] *exilis* - Archey
AIM B6228	tbt	Waikaremoana	[3] est. femur length = 266 mm (B. Gill)
AIM B6261d	fr	Far North, NI	chick, femur length = 65.1 mm
AIM B6666b	fr	Far North, NI	chick, femur length = 77.9 mm
AIM B13978	fr	Far North, NI	chick, femur length = 60.5 mm
CM Av8378	fr	Pyramid Valley	[3] *gravis* - Scarlett
CM Av21330	fr	Takaka	[3] *gravis* - Archey. ID - P. Scofield
CM Av9188	fr	Kapua	[3] Hutton, 1895. ID - T. H. Worthy
CM Av38561	tmt	Gowan Hills Station swamp	est. femur length = 308 mm
OM Av9821	fr	Paerau	[3]
W 1617	fr	Makirikiri	ID - T. H. Worthy

Data for moa bones was obtained from the references indicated in the table notes, or were sourced as described in the Materials and Methods. fr - femur, tbt - tibiotarsus, tmt - tarsometatarsus, NI - North Island.

As a result of being unable to resolve *Euryapteryx* species using set *COI* divergence values we searched for informative single nucleotide polymorphisms (SNPs) within *COI* and control region sequences that could discriminate between possible *Euryapteryx* clades. *COI* SNPs have been used previously to successfully to identify all moa species, with limited resolution however for *Euryapteryx* [13]. Four informative *COI* SNPs were found; one at nucleotide position 7213 (C>T) that separates the two moa samples from the Far North of the North Island from the remaining samples, and three SNPs at positions 7155 (C>T), 7278 (G>A) and 7512 (C>T) that distinguish *Euryapteryx* samples from the south of the South Island from all the others (Table S2). For the mitochondrial control region, a single informative SNP was found in 677 bp of sequence [3]. The SNP occurs at nucleotide position 557 of the *D. robustus* mitochondrial genome (GenBank accession number: AY016013.1). Comparison of 50 *Euryapteryx* sequences from samples from 13 locations on the North Island and 66 samples from 18 locations on the South Island showed that 32 North Island *Euryapteryx* samples carried a derived thymine at position 557 (557T) and 18 carried a cytosine (557C; sequences obtained from [3], [4], [8], [10], [14], [15], [16]. For the South island, all 66 *Euryapteryx* sequences had a cytosine at position 557. This SNP also serves as the defining polymorphism that separates *Euryapteryx* samples belonging to class I (cytosine) from those belonging to class II (thymine) as detemined by eggshell data [10] where the eight thinnest *Euryapteryx* eggshells were associated with the class II 557T SNP (p = 0.004) and the 27 thickest eggshells were associated with the class I 557C SNP (p = <<0.001; Figure 1C). Where skeletal measurements were available (for 23 samples), 11 class II samples had leg bone length measurements that fell within the limits set for *E. curtus curtus* (femora 150–225 mm, tibiotarsi 240–380 mm; [2]) and two, CM Av9243 (femur 235 mm), and AIM B6228 (tibiotarsis 468 mm) that were significantly larger. For class I samples, 8 fell within the leg bone length measurement limits for *E curtus gravis* (femora 215–340 mm, tibiotarsi 405–600 mm; [2] except for AIM B6580 (femur 195 mm) and W 1617 (femur 206 mm). The presence of class I eggshells in the Far North of the North Island was difficult to reconcile considering the absence of bones that could be attributed to *E. curtus gravis* from this area [2]. Further sequencing however of a number of small *Euryapteryx* bones (AIM B6666b, AIM B13978, and AIM B6261d from Tokerau Beach) show that some class I bones at least are present in this region.

## Discussion

Determining species status can be difficult and for some species at least is likely to require a combination of morphological, molecular, physiological, and behavioural data. The absence of substantial morphological, physiological, and behavioural data for extinct animals makes species determination in these animals particularly difficult. The use of a reliable identification tool such as has been provided by *COI* barcode analysis can significantly aid species identification, especially so for extinct animals where DNA can still be extacted from bones tens of thousands of years old.

Using a number of sequences (including the *COI* barcoding region) from several ancient tissues such as bone and eggshell, as well as soil, we have tried to clarify the species status for ancient moa belonging to the genus *Euryapteryx*. For *Euryapteryx, COI* sequences were unable to unequivocably determine species status with seemingly random associations of various samples at a number of different sequence divergence cut-off values. However, the generally large *COI* divergences shown between *Euryapteryx* samples from the south of New Zealand's South Island and the far north of the North Island suggest that these two populations may represent two species. At more than 1.6% divergence, these two populations share a greater divergence than the two recognised species of *Dinornis* as well as between *P. elephantopus* from the South Island and *P. geranoides* from the North Island. The identification of signature *COI* SNPs in these *Euryapteryx* populations suggest they have been isolated for a substantial period. A single control region SNP (557T) found in North Island class II *Euryapteryx* only was also found to be associated with the thinnest *Euryapteryx* eggshells, tentatively suggesting that this SNP may be diagnostic for the proposed *Euryapteryx* subspecies *E. curtus curtus*. Morphological analysis of *Euryapteryx* suggests that *E. curtus curtus* were substantially smaller than *E. curtus gravis* and often had crania with distinct interorbital elevation dorsally rather than the smooth dorsal profile of the latter [2]. The smallest *E. curtus curtus* specimens are those from the late Holocene found in the Far North of New Zealand's North Island [2]. The larger *E. curtus gravis* are proposed to derive from the South Island only, with a population of small stature on Takaka Hill, and the largest in Southland [2]. The identification of *COI* and a control region SNP unique to *Euryapteryx* populations from the Southern South Island and the North Island respectively provide some support for the morphological data.

## Conclusions

The inability to form well-supported clades using either *COI* or control region sequences from central and south North Island and central and north South Island *Euryapteryx* suggest that population structure in these individuals was in a state of flux, possibly as a result of continuing hybridisation events. *Euryapteryx* are unique in this regard, being the only moa that at the molecular level at least does not show distinct South Island/North Island divergence.

## Materials and Methods

### Samples

The moa samples used in this work are shown in Table 1. Moa bone samples W 1617, CM Av38561, AIM B6580, AIM B6228, AIM B6666b, AIM B13978, and AIM B6261d were kindly loaned by the Whanganui Regional Museum (W), Canterbury Museum (CM) and the Auckland Institute and Museum (AIM). Permission to sample moa specimens was obtained from the respective museum curators. No permits were required for the described study, which complied with all relevant regulations.

### Ancient DNA extraction

Approximately 20–50 mg of bone was shaved from the bone surface using a scalpel and incubated at 56°C, with rotation, overnight in 0.3 ml of 0.25 M EDTA/0.01% Triton X100, and ∼0.5 mg of proteinase K. The solution was then cleared by the addition of 75 ul of concentrated HCl. 600 ul of ethanol was then added and the mix was loaded directly onto a Qiagen DNeasy Blood & Tissue Kit silica spin column and washed as recommended by the manufacturers. The DNA was finally eluted from the column with 30 ul of 0.01% Triton X100.

*Ancient DNA precautions*: All DNA extractions were carried out in a physically separate, dedicated ancient DNA laboratory following set criteria [17]. This facility is separated by 500 metres from the main laboratory in another building where amplifications were performed. Most sequences were obtained in both directions from separate amplifications and in most cases from multiple extractions. Sequences from a number of samples were verified by LH at Massey University's Ancient DNA facility in Auckland, New Zealand.

### DNA amplification and sequencing

Approximately 2 ul of DNA was amplified by polymerase chain reaction (PCR) in 10 ul volumes containing 50 mM Tris-Cl pH 8.8, 20 mM (NH_4_)_2_SO_4_, 2.5 mM MgCl_2_, 1 mg/ml BSA, 200 uM each of dGTP, dUTP, dCTP, and dATP, 0.5 uM of each primer, 0.06 U of cod Uracil-DNA Glycosylase (ArcticZymes), and ∼0.3 U of platinum Taq (Invitrogen). The reaction mix was incubated at room temp for approximately 15 min and then subjected to amplification in an ABI Gene amp 9700 thermal cycler using the following parameters: 94°C for 2 min (x 1), 94°C for 20 sec, 56°C for 1 min (x 43). Amplified DNAs were detected by agarose gel electrophoresis in 0.5 x Tris-borate-EDTA buffer (TBE), stained with 50 ng/ml ethidium bromide in 0.5 x TBE, and then visualized over UV light. Positive amplifications were purified by centrifugation through ∼40 ul of dry Sephacryl S200HR and then sequenced in both orientations (using the same primers used for PCR) at the Griffith University DNA Sequencing Facility using Applied Biosystems (ABI) BigDye Terminator v3.1 chemistry and an ABI3730 Genetic Analyzer.

*PCR Amplification primers*: The primers used for amplification of the mitochondrial control region (bases 554–580) and COI barcoding region (bases 6996–7619) are from [10] and [5] respectively. The base numbers shown were determined from the complete mitochondrial genome of *D. robustus*; GenBank accession number AY016013.1. Sequences obtained have been deposited in GenBank (accession numbers: KF888653, KF888654).

### Bioinformatics

Mitochondrial COI and control region sequences were initially aligned in Sequencher (Gene Codes) and then realigned by eye, with minimal gap insertion. Phylogenetic trees were constructed in MEGA 5.05 [18] using Maximum Likelihood parameters and the Tamura-Nei model of nucleotide substitution [20]. Sequence divergences between COI sequences were calculated in MEGA 5.05, [18] using Kimura 2 parameter distance criteria; the standard criteria used for distance estimation for COI sequences [19].

## Supporting Information

Table S1 **Variant COI positions in** ***Euryapteryx*** **.** Numbers correspond to nucleotide position in the complete mitochondrial genome of*D. robustus* (A Y016013.1).
(DOCX)

Table S2 **Estimates of Evolutionary Divergence between COI Sequences for moa.** The number of base substitutions per site between sequences are shown. Analyses were conducted using the Kimura 2-parameter model [1]. The analysis involved 37 nucleotide sequences. Codon positions included were 1st+2nd+3rd+Noncoding. All positions containing gaps and missing data were eliminated. There were a total of 590 positions in the final dataset. Evolutionary analyses were conducted in MEGA 5.05 [2]. Numbers in bold correspond to those representing *Euryapteryx*.
(DOCX)

Table S3 ***Euryapteryx*** **groupings according to percent COI sequence divergence.** Sequences are grouped according to <1.6%, <1.25%, and <0.8% divergence for 596 bp of COI sequence. In each column, one sample (underlined) was compared against all the others. Samples in black meet the indicated divergence level.
(DOCX)

Table S4 **Estimates of Evolutionary Divergence between** ***Apteryx*** **COI Sequences.** The number of base substitutions per site from between sequences are shown. Analyses were conducted using the Kimura 2-parameter model [1]. The analysis involved 18 nucleotide sequences. Codon positions included were 1st+2nd+3rd+Noncoding. All positions containing gaps and missing data were eliminated. There were a total of 619 positions in the final dataset. Evolutionary analyses were conducted in MEGA 5.05 [2]. Am - *Apteryx mantelli*, Arowi - *Apteryx rowi*, Aa - *Apteryx australis*. Numbers in bold show the low COI divergence level between *A. owenii* and *A. haastii*.
(DOCX)
