# Supplementary material for: Complex Species Status for Extinct Moa (Aves: Dinornithiformes) from the Genus Euryapteryx
Source: PLoS One. 2014 Mar 3;9(3):e90212. doi: 10.1371/journal.pone.0090212 (PMC3940832; doi:10.1371/journal.pone.0090212)
Supplement: Table S1 — Variant COI positions in Euryapteryx . Numbers correspond to nucleotide position in the complete mitochondrial genome ofD. robustus (A Y016013.1). (DOCX) [file pone.0090212.s001.docx]

77777777777777777777

00011112222233344555

18914571115701218018

43839503587884935623

AIM B9243 ---G---T-----T-----T

AIM B6595ii -------T-----T------

AIM B6580 -------------T------

AIM B6228 ---------CA-GT------

CM Av21330 G-------G--------C--

CM Av8378 -TC-G---------------

CM Av9188 G--G-T-----A------T-

OM Av9821 G----TT----A---G--T-

CM Av38561 G----T--G--A--A-G-T-

Consensus ACTAACCCATGGACGAATCC

**Table S1. Variant COI positions in *Euryapteryx***. Numbers correspond to nucleotide position in the complete mitochondrial genome of *D. robustus* (AY016013.1).
