# Supplementary material for: Complex Species Status for Extinct Moa (Aves: Dinornithiformes) from the Genus Euryapteryx
Source: PLoS One. 2014 Mar 3;9(3):e90212. doi: 10.1371/journal.pone.0090212 (PMC3940832; doi:10.1371/journal.pone.0090212)
Supplement: Table S2 — Estimates of Evolutionary Divergence between COI Sequences for moa. The number of base substitutions per site between sequences are shown. Analyses were conducted using the Kimura 2-parameter model [1]. The analysis involved 37 nucleotide sequences. Codon positions included were 1st+2nd+3rd+Noncoding. All positions containing gaps and missing data were eliminated. There were a total of 590 positions in the final dataset. Evolutionary analyses were conducted in MEGA 5.05 [2]. Numbers in bold correspond to those representing Euryapteryx. (DOCX) [file pone.0090212.s002.docx]

**CM Av9243 CM Av9243**

**AIM B6595ii 0.00340 AIM B6595ii**

**AIM B6580 0.00511 0.00170 AIM B6580**

**AIM B6228 0.00855 0.00511 0.00340 AIM B6228**

**CM Av8378 0.01201 0.00855 0.00683 0.01027 CM Av8378**

**CM Av21330 0.01201 0.00855 0.00683 0.01027 0.01027 CM Av21330**

**CM Av9188 0.01201 0.01201 0.01027 0.01375 0.01375 0.01027 CM Av9188**

**OM Av9821 0.01724 0.01375 0.01201 0.01549 0.01549 0.01201 0.00511 CM Av9821**

**CM Av38561 0.01900 0.01549 0.01375 0.01724 0.01724 0.01027 0.00683 0.00855 CM Av38561**

CM Av20591 0.04236 0.04236 0.04052 0.03686 0.04052 0.04420 0.04052 0.04605 0.04791 CM Av20591

CM Av34550 0.03329 0.02968 0.02788 0.02431 0.03148 0.03148 0.03148 0.03329 0.03510 0.03869 CM Av34550

CM Av9209 0.04799 0.04428 0.04243 0.04243 0.04986 0.04613 0.04613 0.04799 0.04986 0.04605 0.04243 CM Av9209

AIM B6221 0.06315 0.05932 0.05741 0.05741 0.06123 0.06123 0.06123 0.05932 0.06123 0.05542 0.05741 0.02431 AIM B6221

CM Av21547 0.03323 0.02963 0.02784 0.02784 0.02784 0.02784 0.02784 0.02963 0.03143 0.04598 0.01026 0.04605 0.06112

w1152 0.05741 0.05362 0.05174 0.05174 0.05932 0.05551 0.05551 0.05741 0.05932 0.05731 0.05551 0.01549 0.02968

CM Av21331 0.06123 0.05741 0.05551 0.05551 0.05932 0.05932 0.05932 0.05741 0.05932 0.05353 0.05551 0.02253 0.00170

OM Av4068 0.05932 0.05551 0.05362 0.05362 0.05741 0.05741 0.05362 0.05551 0.05741 0.05353 0.04986 0.01375 0.02788

CM Av9037 0.04236 0.04236 0.04052 0.03686 0.04052 0.04420 0.04052 0.04605 0.04791 0.00000 0.03869 0.04605 0.05542

CM Av8579 0.04236 0.04236 0.04052 0.03686 0.04052 0.04420 0.04052 0.04605 0.04791 0.00000 0.03869 0.04605 0.05542

OM Av10049 0.05542 0.05165 0.04978 0.04978 0.05353 0.05353 0.05353 0.05542 0.05165 0.03499 0.04978 0.05353 0.06883

CM Av36435 0.04236 0.04236 0.04052 0.03686 0.04052 0.04420 0.04052 0.04605 0.04791 0.00340 0.03869 0.04605 0.05542

AIM B6286 0.04799 0.04428 0.04243 0.04243 0.04243 0.04613 0.04613 0.04799 0.04986 0.01373 0.04243 0.04613 0.05741

MNZ 37845 0.05551 0.05174 0.04986 0.04986 0.05362 0.05362 0.05362 0.05551 0.05741 0.05165 0.05362 0.01375 0.02788

CM Av30497 0.04236 0.04236 0.04052 0.03686 0.04052 0.04420 0.04052 0.04605 0.04791 0.00000 0.03869 0.04605 0.05542

MNZ 37844 0.05551 0.05174 0.04986 0.04986 0.05362 0.05362 0.05362 0.05551 0.05741 0.05165 0.05362 0.01375 0.02788

AIM B6310 0.04799 0.04428 0.04243 0.04243 0.04243 0.04613 0.04613 0.04799 0.04986 0.01373 0.04243 0.04613 0.05741

CM SB301 0.04799 0.04428 0.04243 0.04243 0.04986 0.04613 0.04613 0.04799 0.04986 0.04605 0.04243 0.00000 0.02431

w337 0.05741 0.05362 0.05174 0.05174 0.05932 0.05551 0.05551 0.05741 0.05932 0.05731 0.05551 0.01549 0.02968

w1156 0.05741 0.05362 0.05174 0.05174 0.05932 0.05551 0.05551 0.05741 0.05932 0.05731 0.05551 0.01549 0.02968

CM Av8320 0.02609 0.02253 0.02076 0.02076 0.02431 0.02076 0.02076 0.02253 0.02431 0.04605 0.03148 0.04613 0.06507

w336 0.05741 0.05362 0.05174 0.05174 0.05932 0.05551 0.05551 0.05741 0.05932 0.05731 0.05551 0.01549 0.02968

CM Av9132 0.02788 0.02431 0.02253 0.02253 0.02609 0.02253 0.02253 0.02431 0.02609 0.04791 0.03329 0.04799 0.06701

CM Av30495 0.04236 0.04236 0.04052 0.03686 0.04052 0.04420 0.04052 0.04605 0.04791 0.00000 0.03869 0.04605 0.05542

CM Av38563 0.06123 0.05741 0.05551 0.05174 0.05932 0.05932 0.05551 0.05741 0.05932 0.05165 0.04799 0.01549 0.02968

w1284 0.05551 0.05174 0.04986 0.04986 0.05362 0.05362 0.05362 0.05551 0.05741 0.05165 0.05362 0.01375 0.02788

CM Av4139 0.04799 0.04428 0.04243 0.04243 0.04986 0.04613 0.04613 0.04799 0.04986 0.04605 0.04243 0.00000 0.02431

CM Av8927 0.04799 0.04428 0.04243 0.04243 0.04986 0.04613 0.04613 0.04799 0.04986 0.04605 0.04243 0.00000 0.02431

CM Av21547 CM Av21547

w1152 0.05921 w1152

CM Av21331 0.05921 0.02788 CM Av21331

OM Av4068 0.05353 0.02253 0.02609 OM Av4068

CM Av9037 0.04598 0.05731 0.05353 0.05353 CM Av9037

CM Av8579 0.04598 0.05731 0.05353 0.05353 0.00000 CM Av8579

OM Av10049 0.05156 0.06689 0.06689 0.06112 0.03499 0.03499 OM Av10049

CM Av36435 0.04598 0.05731 0.05353 0.05353 0.00340 0.00340 0.03499 CM Av36435

AIM B6286 0.04605 0.05932 0.05551 0.05362 0.01373 0.01373 0.03869 0.01722 AIM B6286

MNZ 37845 0.05731 0.00511 0.02609 0.01724 0.05165 0.05165 0.06112 0.05165 0.05362 MNZ 37845

CM Av30497 0.04598 0.05731 0.05353 0.05353 0.00000 0.00000 0.03499 0.00340 0.01373 0.05165 CM Av30497

MNZ 37844 0.05731 0.00511 0.02609 0.01724 0.05165 0.05165 0.06112 0.05165 0.05362 0.00000 0.05165 MNZ 37844

AIM B6310 0.04605 0.05551 0.05551 0.05362 0.01373 0.01373 0.03869 0.01722 0.00340 0.05362 0.01373 0.05362 AIM B6310

CM SB301 0.04605 0.01549 0.02253 0.01375 0.04605 0.04605 0.05353 0.04605 0.04613 0.01375 0.04605 0.01375 0.04613 CM SB301

w337 0.05921 0.00000 0.02788 0.02253 0.05731 0.05731 0.06689 0.05731 0.05932 0.00511 0.05731 0.00511 0.05551 0.01549

w1156 0.05921 0.00000 0.02788 0.02253 0.05731 0.05731 0.06689 0.05731 0.05932 0.00511 0.05731 0.00511 0.05551 0.01549

CM Av8320 0.02784 0.05551 0.06315 0.05362 0.04605 0.04605 0.04978 0.04605 0.04243 0.05362 0.04605 0.05362 0.04613 0.04613

w336 0.05921 0.00000 0.02788 0.02253 0.05731 0.05731 0.06689 0.05731 0.05932 0.00511 0.05731 0.00511 0.05551 0.01549

CM Av9132 0.02963 0.05741 0.06507 0.05551 0.04791 0.04791 0.05165 0.04791 0.04428 0.05551 0.04791 0.05551 0.04799 0.04799

CM Av30495 0.04598 0.05731 0.05353 0.05353 0.00000 0.00000 0.03499 0.00340 0.01373 0.05165 0.00000 0.05165 0.01373 0.04605

CM Av38563 0.05542 0.02431 0.02788 0.00170 0.05165 0.05165 0.06304 0.05165 0.05551 0.01900 0.05165 0.01900 0.05551 0.01549

w1284 0.05731 0.00511 0.02609 0.01724 0.05165 0.05165 0.06112 0.05165 0.05362 0.00000 0.05165 0.00000 0.05362 0.01375

CM Av4139 0.04605 0.01549 0.02253 0.01375 0.04605 0.04605 0.05353 0.04605 0.04613 0.01375 0.04605 0.01375 0.04613 0.00000

CM Av8927 0.04605 0.01549 0.02253 0.01375 0.04605 0.04605 0.05353 0.04605 0.04613 0.01375 0.04605 0.01375 0.04613 0.00000

w337 w337

w1156 0.00000 w1156

CM Av8320 0.05551 0.05551 CM Av8320

w336 0.00000 0.00000 0.05551 w336

CM Av9132 0.05741 0.05741 0.00170 0.05741 CM Av9132

CM Av30495 0.05731 0.05731 0.04605 0.05731 0.04791 CM Av30495

CM Av38563 0.02431 0.02431 0.05551 0.02431 0.05741 0.05165 CM Av38563

w1284 0.00511 0.00511 0.05362 0.00511 0.05551 0.05165 0.01900 w1284

CM Av4139 0.01549 0.01549 0.04613 0.01549 0.04799 0.04605 0.01549 0.01375 CM Av4139

CM Av8927 0.01549 0.01549 0.04613 0.01549 0.04799 0.04605 0.01549 0.01375 0.00000 CM Av8927

**Table S2. Estimates of Evolutionary Divergence between COI Sequences for moa**. The number of base substitutions per site between sequences are shown. Analyses were conducted using the Kimura 2-parameter model [1]. The analysis involved 37 nucleotide sequences. Codon positions included were 1st+2nd+3rd+Noncoding. All positions containing gaps and missing data were eliminated. There were a total of 590 positions in the final dataset. Evolutionary analyses were conducted in MEGA 5.05 [2]. Numbers in bold correspond to those representing *Euryapteryx*.
