# Supplementary material for: Complex Species Status for Extinct Moa (Aves: Dinornithiformes) from the Genus Euryapteryx
Source: PLoS One. 2014 Mar 3;9(3):e90212. doi: 10.1371/journal.pone.0090212 (PMC3940832; doi:10.1371/journal.pone.0090212)
Supplement: Table S3 — Euryapteryx groupings according to percent COI sequence divergence. Sequences are grouped according to <1.6%, <1.25%, and <0.8% divergence for 596 bp of COI sequence. In each column, one sample (underlined) was compared against all the others. Samples in black meet the indicated divergence level. (DOCX) [file pone.0090212.s003.docx]

**<1.6%**

**CM Av9243 CM Av9243 CM Av9243 CM Av9243 CM Av9243 CM Av9243 CM Av9243 CM Av9243 CM Av9243**

**AIM B6595ii AIM B6595ii AIM B6595ii AIM B6595ii AIM B6595ii AIM B6595ii AIM B6595ii AIM B6595ii AIM B6595ii**

**AIM B6580 AIM B6580 AIM B6580 AIM B6580 AIM B6580 AIM B6580 AIM B6580 AIM B6580 AIM B6580**

**AIM B6228 AIM B6228 AIM B6228 AIM B6228 AIM B6228 AIM B6228 AIM B6228 AIM B6228 AIM B6228**

**CM Av8378 CM Av8378 CM Av8378 CM Av8378 CM Av8378 CM Av8378 CM Av8378 CM Av8378 CM Av8378**

**CM Av21330 CM Av21330 CM Av21330 CM Av21330 CM Av21330 CM Av21330 CM Av21330 CM Av21330 CM Av21330**

**CM Av9188 CM Av9188 CM Av9188 CM Av9188 CM Av9188 CM Av9188 CM Av9188 CM Av9188 CM Av9188**

**OM Av9821 OM Av9821 OM Av9821 OM Av9821 OM Av9821 OM Av9821 OM Av9821 OM Av9821 OM Av9821**

**CM Av38561 CM Av38561 CM Av38561 CM Av38561 CM Av38561 CM Av38561 CM Av38561 CM Av38561 CM Av38561**

**<1.25%**

**CM Av9243 CM Av9243 CM Av9243 CM Av9243 CM Av9243 CM Av9243 CM Av9243 CM Av9243 CM Av9243**

**AIM B6595ii AIM B6595ii AIM B6595ii AIM B6595ii AIM B6595ii AIM B6595ii AIM B6595ii AIM B6595ii AIM B6595ii**

**AIM B6580 AIM B6580 AIM B6580 AIM B6580 AIM B6580 AIM B6580 AIM B6580 AIM B6580 AIM B6580**

**AIM B6228 AIM B6228 AIM B6228 AIM B6228 AIM B6228 AIM B6228 AIM B6228 AIM B6228 AIM B6228**

**CM Av8378 CM Av8378 CM Av8378 CM Av8378 CM Av8378 CM Av8378 CM Av8378 CM Av8378 CM Av8378**

**CM Av21330 CM Av21330 CM Av21330 CM Av21330 CM Av21330 CM Av21330 CM Av21330 CM Av21330 CM Av21330**

**CM Av9188 CM Av9188 CM Av9188 CM Av9188 CM Av9188 CM Av9188 CM Av9188 CM Av9188 CM Av9188**

**OM Av9821 OM Av9821 OM Av9821 OM Av9821 OM Av9821 OM Av9821 OM Av9821 OM Av9821 OM Av9821**

**CM Av38561 CM Av38561 CM Av38561 CM Av38561 CM Av38561 CM Av38561 CM Av38561 CM Av38561 CM Av38561**

**<0.8%**

**CM Av9243 CM Av9243 CM Av9243 CM Av9243 CM Av9243 CM Av9243 CM Av9243 CM Av9243 CM Av9243**

**AIM B6595ii AIM B6595ii AIM B6595ii AIM B6595ii AIM B6595ii AIM B6595ii AIM B6595ii AIM B6595ii AIM B6595ii**

**AIM B6580 AIM B6580 AIM B6580 AIM B6580 AIM B6580 AIM B6580 AIM B6580 AIM B6580 AIM B6580**

**AIM B6228 AIM B6228 AIM B6228 AIM B6228 AIM B6228 AIM B6228 AIM B6228 AIM B6228 AIM B6228**

**CM Av8378 CM Av8378 CM Av8378 CM Av8378 CM Av8378 CM Av8378 CM Av8378 CM Av8378 CM Av8378**

**CM Av21330 CM Av21330 CM Av21330 CM Av21330 CM Av21330 CM Av21330 CM Av21330 CM Av21330 CM Av21330**

**CM Av9188 CM Av9188 CM Av9188 CM Av9188 CM Av9188 CM Av9188 CM Av9188 CM Av9188 CM Av9188**

**OM Av9821 OM Av9821 OM Av9821 OM Av9821 OM Av9821 OM Av9821 OM Av9821 OM Av9821 OM Av9821**

**CM Av38561 CM Av38561 CM Av38561 CM Av38561 CM Av38561 CM Av38561 CM Av38561 CM Av38561 CM Av38561**

**Table S3. *Euryapteryx* groupings according to percent COI sequence divergence**. Sequences are grouped according to <1.6%, <1.25%, and <0.8% divergence for 596bp of COI sequence. In each column, one sample (underlined) was compared against all the others. Samples in black meet the indicated divergence level.
