# Supplementary material for: Complex Species Status for Extinct Moa (Aves: Dinornithiformes) from the Genus Euryapteryx
Source: PLoS One. 2014 Mar 3;9(3):e90212. doi: 10.1371/journal.pone.0090212 (PMC3940832; doi:10.1371/journal.pone.0090212)
Supplement: Table S4 — Estimates of Evolutionary Divergence between Apteryx COI Sequences. The number of base substitutions per site from between sequences are shown. Analyses were conducted using the Kimura 2-parameter model [1]. The analysis involved 18 nucleotide sequences. Codon positions included were 1st+2nd+3rd+Noncoding. All positions containing gaps and missing data were eliminated. There were a total of 619 positions in the final dataset. Evolutionary analyses were conducted in MEGA 5.05 [2]. Am - Apteryx mantelli, Arowi - Apteryx rowi, Aa - Apteryx australis. Numbers in bold show the low COI divergence level between A. owenii and A. haastii. (DOCX) [file pone.0090212.s004.docx]

**Ahaastii FT2920 AhFT2920**

**Ahaastii CD830 0.00162 AhCD830**

**Ahaastii FT2922 0.00000 0.00162 AhFT2922**

**Ahaastii LH 0.00486 0.00324 0.00486 AhLH**

**Aowenii O-20578 0.01638 0.01471 0.01638 0.01802 AoO-20578**

**Aowenii WS1764 0.01804 0.01638 0.01804 0.01969 0.00162 AoWS1764**

**Aowenii O-20599 0.01638 0.01471 0.01638 0.01802 0.00000 0.00162 AoO-20599**

**Aowenii LH 0.01969 0.01802 0.01969 0.01471 0.00324 0.00486 0.00324 AoLH**

Am GS20 0.07070 0.06885 0.07070 0.06875 0.07070 0.07256 0.07070 0.07060 AaGS20

Am CD888 0.07070 0.06885 0.07070 0.06875 0.07070 0.07256 0.07070 0.07060 0.00000 AaCD888

Am BK1 0.07256 0.07070 0.07256 0.07060 0.07256 0.07442 0.07256 0.07245 0.00162 0.00162 AaBK1

Am CD890 0.06701 0.06518 0.06701 0.06508 0.06701 0.06885 0.06701 0.06691 0.00324 0.00324 0.00487 AaCD890

Am LH 0.07793 0.07606 0.07793 0.07245 0.07793 0.07980 0.07793 0.07431 0.00649 0.00649 0.00812 0.00976 AaLH

Arowi R34152 0.05791 0.05610 0.05791 0.05963 0.05791 0.05971 0.05791 0.06144 0.01642 0.01642 0.01809 0.01642 0.02308

Arowi R28242 0.05791 0.05610 0.05791 0.05963 0.05791 0.05971 0.05791 0.06144 0.01642 0.01642 0.01809 0.01642 0.02308

Arowi R34155 0.05791 0.05610 0.05791 0.05963 0.05791 0.05971 0.05791 0.06144 0.01642 0.01642 0.01809 0.01642 0.02308

Arowi LH 0.06144 0.05963 0.06144 0.05610 0.06144 0.06326 0.06144 0.05791 0.01640 0.01640 0.01807 0.01640 0.01974

Aa LH 0.05791 0.05610 0.05791 0.05260 0.06135 0.06317 0.06135 0.05782 0.02139 0.02139 0.02308 0.02139 0.02477

Arowi R34152 ArR34152

Arowi R28242 0.00000 ArR28242

Arowi R34155 0.00000 0.00000 ArR34155

Arowi LH 0.00324 0.00324 0.00324 AokLH

Aa LH 0.02139 0.02139 0.02139 0.01807 AtLH

**Table S4. Estimates of Evolutionary Divergence between Apteryx COI Sequences**. The number of base substitutions per site between sequences are shown. Analyses were conducted using the Kimura 2-parameter model [1]. The analysis involved 18 nucleotide sequences. Codon positions included were 1st+2nd+3rd+Noncoding. All positions containing gaps and missing data were eliminated. There were a total of 619 positions in the final dataset. Evolutionary analyses were conducted in MEGA 5.05 [2]. Am - *Apteryx mantelli*, Arowi - *Apteryx rowi*, Aa - *Apteryx australis*. Numbers in bold show the low COI divergence level between *A. owenii* and *A. haastii*.
